# Long-Term Weight Change and Glycemic Control in Patients With Type 2 Diabetes Mellitus and Treated vs. Untreated Sleep-Disordered Breathing—Analysis From the DIAbetes COhoRtE

**DOI:** 10.3389/fneur.2021.745049

**Published:** 2021-12-02

**Authors:** Louisa Schaller, Michael Arzt, Bettina Jung, Carsten A. Böger, Iris M. Heid, Stefan Stadler

**Affiliations:** ^1^Department of Internal Medicine II, University Hospital Regensburg, Regensburg, Germany; ^2^Department of Nephrology, University Hospital Regensburg, Regensburg, Germany; ^3^Department of Genetic Epidemiology, University Hospital Regensburg, Regensburg, Germany

**Keywords:** glycemic control (HbA1c), positive airway pressure (PAP), sleep-disordered breathing, weight, type 2 diabetes mellitus

## Abstract

**Hypothesis:** Positive airway pressure (PAP) is the standard treatment for sleep-disordered breathing (SDB), a prevalent condition in patients with type 2 diabetes mellitus (DM2). Recent studies showed that short-term PAP treatment may cause weight gain. However, long-term data for patients with DM2 are scarce. Therefore, the aim of the present analysis was to assess changes in weight and glycemic control in patients with DM2 and treated vs. untreated SDB.

**Methods:** The DIAbetes COhoRtE (DIACORE) study is a prospective population-based cohort study in patients with DM2. At baseline, patients of the DIACORE-SDB sub-study were tested for SDB [defined as apnea-hypopnea-index (AHI) ≥ 15/h] using a two-channel ambulatory SDB-monitoring device. In this observational study, PAP treatment was initiated in a subgroup of patients with SDB (SDB PAP) within clinical routine between the baseline and first follow-up visit [median observation period of 2.3 (2.2; 2.4) years], whereas the other patients with SDB did not receive PAP (SDB untreated). At baseline and first follow-up visit, weight and HbA1c were assessed.

**Results:** Of the 346 patients with SDB [mean age 68 years, 71% male, body-mass index (BMI) 31.9 kg/m^2^], 17% were in the SDB PAP and 83% in the SDB untreated group. Weight change within the observation period was similar in both groups (−0.2 and −0.9 kg; *p* = 0.322). The percentage of patients with severe weight gain (≥ 5 kg) within the observation period was significantly higher in the SDB PAP group compared to the SDB untreated group (15.0 vs. 5.6%; *p* = 0.011). Multivariable regression analysis, accounting for baseline HbA1c, insulin substitution, BMI, waist-to-hip ratio (WHR), physical activity, and AHI, showed that PAP treatment was significantly associated with a weight gain ≥ 5 kg [odds ratio (OR) = 3.497; 95% CI (1.343; 9.106); *p* = 0.010] and an increase in HbA1c [B = 2.410; 95% CI (0.118; 4.702); *p* = 0.039].

**Conclusion:** Median weight change was similar in patients with SDB with and without PAP treatment. However, patients with DM2 and PAP treatment have an increased risk of severe long-term weight gain and an increase in HbA1c.

**Clinical Trial registration:** DRKS00010498

## Introduction

Sleep-disordered breathing (SDB) is a highly prevalent condition in patients with type 2 diabetes mellitus (DM2) (1). The prevalence of at least moderate obstructive sleep apnea [OSA, defined as apnea-hypopnea-index (AHI) ≥ 15/h] is estimated as 25–59% in patients with DM2 (2), compared to a prevalence of 50% in men and 23% in women in the general population (3). OSA is characterized by recurrent pharyngeal collapse, which results in upper airway obstruction and intermittent hypoxia (4). Arousals from sleep restore the patency of the airway but lead to sleep fragmentation (4).

Obesity is one of the most important risk factors for OSA (4). It contributes to the development of OSA because pharyngeal fat deposition reduces upper airway lumen and fat deposition in the thorax and abdomen reduces lung volume (5). Furthermore, elevated levels of leptin and inflammatory cytokines in obese patients impair the respiratory drive and the neuromuscular control of the upper airway (5).

The interaction between OSA and obesity is bidirectional since OSA can facilitate weight gain (6). Many patients with OSA suffer from daytime sleepiness due to sleep fragmentation. Therefore, they may be less active and gain weight if caloric intake is not reduced accordingly (7). Moreover, leptin and ghrelin levels are increased in patients with OSA (8). Leptin is produced by adipocytes and inhibits appetite (9). However, permanently elevated leptin levels in patients with OSA lead to a resistance to the weight-reducing effects of leptin (10). Elevated ghrelin levels in patients with OSA stimulate appetite, which can result in higher food intake followed by weight gain (7). These alterations in appetite-regulating hormones in addition to the activation of the sympathetic nervous system, the stimulation of the hypothalamic-pituitary-adrenal axis, and the increase in systemic inflammation are possible mechanisms for developing insulin resistance and DM2 in patients with OSA (11). Thus, OSA, obesity, and DM2 influence and impair each other (11). This is of particular interest as OSA, obesity, and DM2 are important cardiovascular risk factors (12).

There are some possible therapies of OSA, such as mandibular advancement devices, active position therapy, and upper airway stimulation therapy (13), but positive airway pressure (PAP) is the standard first-line treatment for OSA (14). It delivers compressed air into the upper airway to keep it open (14). Hence, the number of apneas and hypopneas and subjective daytime sleepiness are reduced (14). In meta-analyses, short-term PAP did not improve the glycemic control measured by HbA1c in patients with DM2 (15–19). The effect of PAP on weight was controversial. Some studies reported weight gain (20, 21), while others reported weight loss after initiation of PAP treatment (22). Drager et al. showed in a meta-analysis that PAP treatment promotes a small but significant increase in weight and BMI (23). However, data for patients with DM2 are scarce. The previous meta-analyses included only 128 and 496 patients with DM2 and analyzed only the short-term effect of PAP (treatment duration ≤ 6 months) on BMI (15, 18). To our knowledge, so far there are no studies investigating the long-term weight change following PAP treatment specifically in patients with DM2. Therefore, the aim of the present analysis was to assess long-term changes in weight and glycemic control in patients with DM2 and treated vs. untreated SDB.

## Methods

### Study Design—DIAbetes COhoRtE and DIACORE-SDB Sub-Study

The analyzed patients were participants of the DIACORE-SDB sub-study. DIACORE (DRKS00010498) is a prospective cohort study with follow-up visits every 2 years in patients with DM2 (24). The baseline study was conducted from 2010 to 2014 and included 3,000 patients recruited in the two study centers Regensburg and Mannheim, as previously described (24, 25). Briefly, written invitations were mailed to all patients with DM2 who were registered with one of five medical insurance companies and two diabetologists in Regensburg (24, 25). Additional invitations were sent to patients with DM2 who had received inpatient treatment at the University Hospital Regensburg's Internal Medicine Departments within 2 years before the mailing (24, 25).

**Figure 1:**
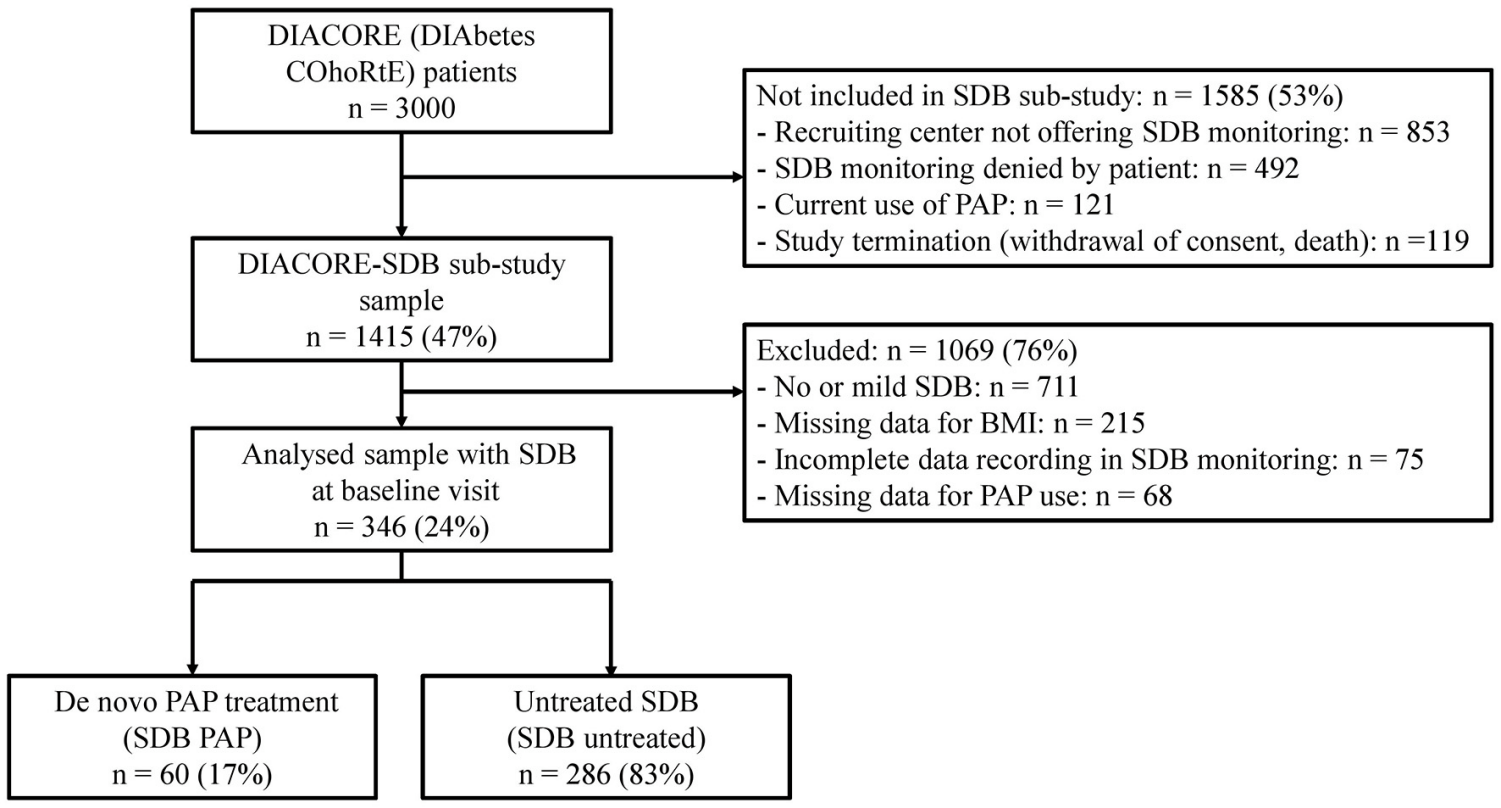
Study flow chart. BMI, body-mass index; PAP, positive airway pressure; SDB, sleep-disordered breathing.

Of the 3,000 patients recruited at baseline, we invited all patients recruited in Regensburg into the DIACORE-SDB sub-study. Total 1,415 patients agreed to participate and were tested with a two-channel ambulatory SDB-monitoring device at the baseline visit (Figure 1). Monitoring for SDB was not performed in the Mannheim study center (25). The patients did not receive any lifestyle recommendations within the study. The protocol, data protection strategy, and study procedures were approved by the Ethics Committees of participating institutions and are in accordance with the Declaration of Helsinki. Patients participated in DIACORE only after providing informed written consent. The study protocol has been previously described (24, 25).

### Study Population—DIACORE and DIACORE-SDB Sub-Study

All patients with DM2 living in the region around Regensburg and Speyer were eligible for participating in DIACORE (24). The inclusion criteria were age ≥18 years, self-reported Caucasian ethnicity, and prevalent DM2 (24). Furthermore, patients were only included if they were able to fully understand the study information and provide informed written consent (24).

Exclusion criteria were the following: chronic renal replacement therapy, history of active malignancy within the last 5 years, autoimmune disease potentially affecting kidney function, hemochromatosis, type 1 diabetes, acute infection, fever, pregnancy, and chronic viral hepatitis or HIV infection (24). Patients from Regensburg were included in the DIACORE-SDB sub-study if they consented to perform SDB monitoring. Patients with current use of PAP were excluded (25).

### Assessments at Baseline and Follow-Up Visit—DIACORE and DIACORE-SDB Sub-Study

**Figure 2:**
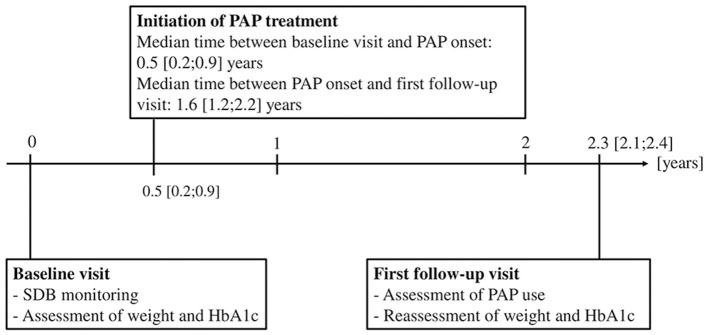
Timeline of initiation of PAP treatment and assessment of clinical data within the median observation period of 2.3 (2.2; 2.4) years. The mean self-reported PAP usage was 6.2 ± 2.4 h/night. Total 87% of the patients in the SDB PAP group used their PAP device ≥4 h/night, 8% did not use their PAP device regularly. HbA1c, hemoglobin A1c; PAP, positive airway pressure; SDB, sleep-disordered breathing.

A standardized protocol, such as physical examination, blood sampling, and a standardized online questionnaire, was performed at baseline and first follow-up visit (24). Anthropometric parameters (weight, height, waist circumference, and hip circumference) were measured in light clothing without shoes (24). Whole blood samples were drawn after the patient had rested for at least 15 min (24). Medication, physical activity level, and alcohol and nicotine consumption were determined by a questionnaire (24). High alcohol consumption was defined as drinking alcohol more than two times a week. Nicotine consumption was assessed by questionnaire and dichotomized as current vs. non-current smoking. Patients who have never smoked were included in the non-current smoking group. Low physical activity was defined as exercise less than three times a week (26). Severe weight gain within the observation period was defined as weight gain ≥5 kg or an increase in BMI ≥2 kg/m^2^ (27). In the DIACORE-SDB sub-study, SDB was assessed at the baseline visit, the initiation of PAP treatment was assessed at the first follow-up visit (Figure 2). In this analysis, the observation period was defined as the time between baseline and first follow-up visit.

### Assessment of SDB at Baseline Visit—DIACORE-SDB Sub-Study

Nasal flow and pulse oximetry were measured using the portable ApneaLink® device (ResMed, Sydney, Australia) as previously described (25). Trained study personnel instructed participants on how to use the device at home. The use of ApneaLink® for assessing SDB has been validated in several studies (28, 29). By comparing ApneaLink® to polysomnography, the gold standard in assessing SDB, studies have reported a sensitivity of 73–91% and a specificity of 87–95% using an AHI cut-off value of 15/h (29, 30). AHI, oxygen desaturation index, mean oxygen saturation, and minimum oxygen saturation were documented (25). The default settings of the monitoring device were used for the definitions of apnea and hypopnea. Apnea was defined as an 80% decrease in airflow for 10 s, and hypopnea was defined as a decrease in the airflow by 50–80% vs. baseline for 10 s followed by a 4% decrease in oxygen saturation (31). ApneaLink® data were automatically scored but manually checked for accuracy and plausibility. Clinically relevant SDB was defined as AHI ≥ 15/h (32). Due to the missing chest band, a clear differentiation in obstructive and central apnea was not possible.

Additionally, subjective daytime sleepiness was assessed using the self-administered, validated Epworth Sleepiness Scale. Individuals were asked to rate their likelihood of falling asleep in several common situations. Possible scores range from 0 to 24. Excessive daytime sleepiness was defined as a score of ≥11 (33).

### Assessment of PAP Treatment at First Follow-Up Visit – DIACORE-SDB Sub-Study

Results of the SDB monitoring at the baseline visit were reported to the patient and if requested, to their general practitioner. In the DIACORE-SDB sub-study, no treatment recommendation in respect of SDB was given to the patients. The decision to send the patient to a sleep laboratory for further diagnosis and treatment of SDB was made by the patient and general practitioner.

A newly initiated PAP treatment was assessed by questionnaire at the first follow-up visit (Figure 2). If a patient claimed to have started PAP treatment within the observation period, the start of treatment and the self-reported usage (hours used per night and days used per week) were recorded. The mean self-reported usage per night was calculated using the formula $\frac{\left(hours\;used\;per\;night\;\times \;days\;used\;per\;week\right)}{7\;days}$.

### Statistical Analysis

Descriptive data were represented as mean ± SD for normally distributed variables and as median with interquartile ranges for non-normally distributed variables. Differences in continuous variables were evaluated with unpaired *t*-test for normally distributed variables and with Mann-Whitney *U*-test for non-normally distributed variables. Categorical variables were compared by a chi-square test and baseline and follow-up data by paired *t*-test.

Linear regression analysis was performed for continuous variables and logistic regression analysis for categorical variables to analyze the association of PAP treatment with change in HbA1c and weight gain ≥5 kg, respectively. Age, sex, HbA1c, oral antidiabetic drugs, insulin substitution, BMI, waist-to-hip ratio (WHR), physical activity, smoking status, alcohol consumption, Epworth Sleepiness Scale, and AHI at baseline were used as covariates. All variables with a *p*-value <0.2 in univariable regression were included in multivariable regression analysis (34). In addition, a multivariable regression with known potential confounders of change in weight and HbA1c, including baseline HbA1c, insulin substitution, BMI, WHR, physical activity, AHI, and PAP treatment, was performed. Results were shown as beta estimates with 95% CI in linear regression models and odds ratio (OR) with 95% CI in logistic regression models. The *p*-values < 0.05 were considered statistically significant. Data were analyzed using the SPSS software package (SPSS 25.0, IBM SPSS Statistics, Armonk, NW, USA).

## Results

Of the 1,415 patients participating in the DIACORE-SDB sub-study, 346 had clinically relevant SDB (AHI ≥ 15/h) at baseline and were included in the analysis (Figure 1). At baseline, the mean age was 68 years, and 71% were men. The patients were mostly obese (mean BMI 31.9 kg/m^2^), had an HbA1c of 52 ± 11 mmol/mol (6.9 ± 1.0%), and a median DM2 duration of 11 (6, 17) years (Table 1).

**Table 1:** Clinical characteristics of the 346 analyzed subjects at the DIACORE baseline visit.

	**Total sample**	**SDB PAP**	**SDB untreated**	***p*-value**
*n* (%)	346 (100.0)	60 (17.3)	286 (82.7)	
Age (years)	68.2 ± 7.4	66.4 ± 7.5	68.6 ± 7.3	**0.037**
Sex (male), *n* (%)	247 (71.4)	46 (76.7)	201 (70.3)	0.320
BMI (kg/m^2^)	31.9 ± 5.5	32.2 ± 4.4	31.9 ± 5.8	0.721
Weight (kg)	92.1 ± 17.9	93.0 ± 14.1	91.9 ± 18.6	0.661
Waist-hip ratio (%)	97 ± 7	99 ± 7	97 ± 7	**0.011**
Diabetes duration (years)	10.9 (6.4; 17.2)	11.0 (5.7; 22.5)	10.9 (6.6; 17.0)	0.992
HbA1c (mmol/mol)	52 ± 11	50 ± 9	53 ± 12	0.075
HbA1c (%)	6.9 ± 1.0	6.7 ± 0.8	7.0 ± 1.1	0.075
Oral antidiabetic drugs, *n* (%)	263 (76.0)	48 (80.0)	215 (75.2)	0.426
Insulin substitution, *n* (%)	127 (36.7)	18 (30.0)	109 (38.1)	0.236
Systolic blood pressure (mmHg)	141 ± 18	140 ± 19	142 ± 18	0.499
Diastolic blood pressure (mmHg)	74 ± 10	73 ± 11	74 ± 10	0.451
Current smoking, *n* (%)	14 (4.0)	1 (1.7)	13 (4.5)	0.304
High alcohol consumption, *n* (%)	123 (35.5)	21 (35.0)	102 (35.7)	0.922
Low physical activity, *n* (%)	200 (57.8)	36 (60.0)	164 (57.3)	0.705
Apnea-hypopnea-index (/h)	23 (18, 33)	28 (20, 49)	22 (18, 30)	**0.001**
Nocturnal SpO_2_ <90% (min)	79 (39; 182)	88 (43; 174)	79 (38; 187)	0.708
Epworth Sleepiness Scale	5.0 (3.0; 7.0)	6.0 (3.0; 8.0)	5.0 (3.0; 7.0)	**0.043**
Excessive daytime sleepiness, *n* (%)	22 (6.4)	4 (6.7)	18 (6.3)	0.900

*Results are provided as mean ± SD for normally distributed variables and as median with interquartile ranges for non-normally distributed variables*.

*DIACORE, DIAbetes COhoRtE; BMI, body-mass index; HbA1c, hemoglobin A1c; PAP, positive airway pressure; SDB, sleep-disordered breathing; SpO_2_, peripheral oxygen saturation. Excessive daytime sleepiness = Epworth Sleepiness Scale ≥11; high alcohol consumption = drinking alcohol > 2 × /week; low physical activity = exercise <3 × /week*.

*The bold values are p-values < 0.05*.

Within the observation period of 2.3 (2.1; 2.4) years, 60 patients (17%) initiated PAP treatment (SDB PAP), while 286 patients (83%) with SDB did not start PAP (SDB untreated, Figure 1). Thus, the cohort of 346 SDB patients was divided into the SDB PAP group and the SDB untreated group. The PAP treatment was initiated at 0.5 (0.2; 0.9) years after the baseline visit, and the median duration between initiation of PAP treatment and the first follow-up visit was 1.6 (1.2; 2.2) years (Figure 2). The percentage of patients who used PAP for at least 1 year was 81%. The mean self-reported PAP usage was 6.2 ± 2.4 h per night and 52 patients (87%) used their PAP device ≥4 h per night.

The patients of the SDB PAP group had a higher WHR at baseline than the patients of the SDB untreated group (*p* = 0.011), but there was no difference in baseline weight or BMI (Table 1). Furthermore, the patients of the SDB PAP group were younger and had a higher baseline AHI compared to the patients of the untreated SDB group (*p* = 0.037 and *p* = 0.001; Table 1).

### Weight Change Within the Observation Period

After a median observation period of 2.3 (2.1; 2.4) years, there was a statistically significant decrease in weight and BMI compared to baseline in the SDB untreated group (each *p* < 0.001), but not in the SDB PAP group (Table 2). However, the changes in weight and BMI within the observation period were similar in the SDB PAP and SDB untreated groups (Table 2; Supplementary Figure 1A). In the SDB PAP group, the percentage of patients with severe weight gain ≥5 kg within the observation period was significantly higher than in the SDB untreated group (*p* = 0.011, Supplementary Figure 1B). Moreover, the percentage of patients with a relevant increase in BMI (defined as ≥ 2 kg/m^2^) within the observation period was significantly higher in the SDB PAP group than in the SDB untreated group (*p* = 0.029; Table 2).

**Table 2:** Changes in weight and HbA1c of the analyzed 346 subjects within the observation period of 2.3 (2.2;2.4) years.

	**Total sample**	**SDB PAP**	**SDB untreated**	***p*-value**
*n* (%)	346 (100)	60 (17.3)	286 (82.7)	
Weight change (kg)	−0.8(−3.2; 1.3)^a^	−0.2 (−3.2; 2.6)	−0.9 (−3.2; 1.3)	0.322
Weight gain ≥ 5 kg, *n* (%)	25 (7.2)	9 (15.0)	16 (5.6)	**0.011**
Weight loss ≥ 5 kg, *n* (%)	50 (14.5)	8 (13.3)	42 (14.7)	0.787
Change in BMI (kg/m^2^)	−0.3 (−1.1; 0.5)^a^	−0.1 (−1.1; 0.9)	−0.3 (−1.1; 0.5)^a^	0.353
Increase in BMI ≥ 2 kg/m^2^, *n* (%)	16 (4.6)	6 (10.0)	10 (3.5)	**0.029**
Decrease in BMI ≥ 2 kg/m^2^, *n* (%)	40 (11.6)	7 (11.7)	33 (11.5)	0.977
Change in HbA1c (mmol/mol)	1 (−4; 5)	3 (−2; 7)^a^	0 (−4; 5)	**0.020**
Change in HbA1c (%)	0.1 (−0.4; 0.5)	0.3 (−0.2; 0.7)^a^	0.0 (−0.4; 0.5)	**0.023**
Increase in HbA1c ≥ 1%, *n* (%)	30 (8.7)	7 (11.7)	23 (8.1)	0.369
Decrease in HbA1c ≥ 1%, *n* (%)	29 (8.4)	2 (3.3)	27 (9.5)	0.119

*Results are provided as median with interquartile ranges for continuous variables and absolute value (%) for categorical variables*.

*BMI, body-mass index; HbA1c, hemoglobin A1c; PAP, positive airway pressure; SDB, sleep-disordered breathing*.

a *Significant change to baseline*.

*The bold values are p-values < 0.05*.

In the univariable regression analysis, PAP treatment, WHR, and HbA1c at baseline were significantly associated with severe weight gain within the observation period (Table 3). Multivariable regression including PAP treatment, WHR, and HbA1c, at baseline showed that PAP treatment and HbA1c at baseline were associated with an increased OR for a severe weight gain ≥5 kg within the observation period [OR = 3.256; 95% CI (1.293; 8.023); *p* = 0.012 and OR = 1.036; 95% CI (1.006; 1.066); *p* = 0.017, respectively; Table 3].

**Table 3:** Modulators for weight gain ≥5 kg and change in HbA1c within the observation period in 346 patients with type 2 diabetes mellitus^*^.

	**Weight gain** **≥** **5 kg**				**Change in HbA1c (mmol/mol)**			
	**Univariable analysis**		**Multivariable analysis**		**Univariable analysis**		**Multivariable analysis**	
	**OR (95% CI)**	***p*-value**	**OR (95% CI)**	***p*-value**	**B (95% CI)**	***p*-value**	**B (95% CI)**	***p*-value**
Age (years)	0.978 (0.928; 1.032)	0.423			−0.027 (−0.150; 0.096)	0.666		
Sex	0.453 (0.151; 1.356)	0.157			−0.736 (−2.755; 1.283)	0.474		
HbA1c (mmol/mol)	1.029 (1.001; 1.058)	**0.044**	1.036 (1.006; 1.066)	**0.017**	−0.284 (−0.358; −0.209)	** <0.001**	−0.277 (−0.351; −0.202)	** <0.001**
Oral antidiabetic drugs (yes vs. no)	1.714 (0.571; 5.143)	0.337			−0.639 (−2.770; 1.491)	0.556		
Insulin substitution (yes vs. no)	1.164 (0.506; 2.671)	0.723			0.523 (−1.366; 2.411)	0.587		
BMI (kg/m^2^)	1.024 (0.955; 1.097)	0.504			−0.057 (−0.221; 0.107)	0.494		
Waist-hip ratio (%)	1.068 (1.006; 1.134)	**0.032**	1.059 (0.994; 1.127)	0.075	0.039 (−0.090; 0.168)	0.552		
Low physical activity	1.324 (0.568; 3.085)	0.516			0.525 (−1.320; 2.370)	0.576		
Current smoking	0.987 (0.124; 7.870)	0.990			−0.039 (−4.656; 4.579)	0.987		
High alcohol consumption	1.021 (0.437; 2.385)	0.961			0.171 (−1.731; 2.073)	0.860		
Epworth sleepiness scale	1.021 (0.908; 1.148)	0.731			−0.161 (−0.431; 0.108)	0.240		
Apnea-hypopnea-index (/h)	1.002 (0.972; 1.032)	0.921			0.024 (−0.045; 0.092)	0.497		
PAP treatment (yes vs. no)	2.978 (1.248; 7.106)	**0.014**	3.256 (1.293; 8.023)	**0.012**	3.074 (0.692; 5.455)	**0.012**	2.276 (0.047; 4.505)	**0.045**

*Shown are the unstandardized regression coefficients by regression analysis, 95% CI and p-values*.

*BMI, body-mass index; CI, confidence interval; HbA1c, hemoglobin A1c; PAP, positive airway pressure; SDB, sleep-disordered breathing. High alcohol consumption = drinking alcohol > 2 × /wk; low physical activity = exercise <3 × /wk*.

* *All variables with a p-value <0.2 in univariable regression were included in multivariable regression analysis (34)*.

*The bold values are p-values <0.05*.

In addition, we performed a multivariable regression model, including known potential modulators of weight change, i.e., baseline HbA1c, insulin substitution, BMI, WHR, physical activity, AHI, and PAP treatment (Table 4). Also in this model, PAP treatment and HbA1c were significantly associated with severe weight gain ≥5 kg within the observation period [OR = 3.497; 95% CI (1.343; 9.106); *p* = 0.010 and OR = 1.036; 95% CI (1.006; 1.068); *p* = 0.019, respectively; Table 4]. Including current smoking as another confounder into this model did not change the association between PAP treatment and severe weight gain ≥5 kg within the observation period significantly [OR = 3.500; 95% CI (1.337; 9.163); *p* = 0.011].

**Table 4:** Multivariable regression analysis with known potential modulators for weight gain ≥5 kg and change in HbA1c within the observation period in 346 patients with type 2 diabetes mellitus.

	**Weight gain** **≥** **5 kg**		**Change in HbA1c (mmol/mol)**	
	**OR (95% CI)**	***p*-value**	**B (95% CI)**	***p*-value**
HbA1c (mmol/mol)	1.036 (1.006; 1.068)	**0.019**	−0.297 (−0.375; −0.220)	** <0.001**
Insulin substitution (yes vs. no)	0.896 (0.362; 2.222)	0.813	2.374 (0.554; 4.194)	**0.011**
BMI (kg/m^2^)	0.999 (0.914; 1.093)	0.989	−0.016 (−0.179; 0.146)	0.845
Waist-hip ratio (%)	1.064 (0.996; 1.137)	0.066	0.022 (−0.101; 0.146)	0.720
Low physical activity	1.534 (0.618; 3.807)	0.356	0.349 (−1.378; 0.190)	0.691
Apnea-hypopnea-index (/h)	0.987 (0.955; 1.021)	0.455	−0.005 (−0.071; 0.062)	0.893
PAP treatment (yes vs. no)	3.497 (1.343; 9.106)	**0.010**	2.410 (0.118; 4.702)	**0.039**

*Shown are the unstandardized regression coefficients by regression analysis, 95% CI and p-values*.

*BMI, body-mass index; HbA1c, hemoglobin A1c; PAP, positive airway pressure; SDB, sleep-disordered breathing; low physical activity = exercise <3 × /wk*.

*The bold values are p-values < 0.05*.

In a sub-analysis stratified by sex, the higher percentage of patients with severe weight gain within the observation period in the SDB PAP group remained significant among men (19.6 vs. 6.0% in the untreated SDB group, *p* = 0.003). In women, there was no significant difference between the SDB PAP and SDB untreated groups (0.0 vs. 4.7%, *p* = 0.407). The association of severe weight gain with PAP treatment was not significantly different between men and women, accounting for known modulators for weight gain, such as baseline HbA1c, insulin substitution, BMI, WHR, physical activity, and AHI (interaction term, *p* = 0.999).

### Changes in Glycemic Control Within the Observation Period

The increase in HbA1c within the median observation period of 2.3 (2.2; 2.4) years was significantly higher in the SDB PAP group than in the SDB untreated group (Table 2; Supplementary Figure 1C). Moreover, in the SDB PAP group, the HbA1c was increased significantly compared to baseline (*p* = 0.008). There was no significant difference between the SDB PAP and SDB untreated groups regarding a strong increase (≥1%) or a strong decrease (≥1%) in HbA1c within the observation period (Table 2).

In univariable and multivariable regression analyses, adjusting for PAP treatment and HbA1c, PAP treatment, and HbA1c at baseline were significantly associated with changes in HbA1c (Table 3). PAP treatment was a predictor for an increase in HbA1c within the observation period [B = 2.276; 95% CI (0.047; 4.505); *p* = 0.045; Table 3].

Additionally, we performed a multivariable regression model, including known potential modulators of change in HbA1c, i.e., baseline HbA1c, insulin substitution, BMI, WHR, physical activity, AHI, and PAP treatment (Table 4). PAP treatment was significantly associated with an increase in HbA1c within the observation period [B = 2.410; 95% CI (0.118; 4.702); *p* = 0.039; Table 4].

In men, the increase in HbA1c within the observation period was significantly higher in the SDB PAP group compared to the SDB untreated group [3 mmol/mol (0.3%) vs. 0 mmol/mol (0.0%), *p* = 0.017], whereas in women, there was no difference between the SDB PAP and the SDB untreated groups [2 mmol/mol (0.2%) vs. 0 mmol/mol (0.0%), *p* = 0.388]. The association of change in HbA1c with PAP treatment was not significantly different between men and women accounting for known modulators for change in HbA1c, such as baseline HbA1c, insulin substitution, BMI, WHR, physical activity, and AHI (interaction term, *p* = 0.505).

## Discussion

The present analysis investigated changes in weight and glycemic control in patients with DM2 and treated vs. untreated SDB and resulted in several key findings. First, there was no significant association between PAP treatment and median weight change within the observation period. Second, the percentage of patients with a severe weight gain ≥5 kg within the observation period was significantly higher in patients of the SDB PAP group. The SDB PAP group had a 3.5-fold increased OR for a weight gain ≥5 kg compared to the SDB untreated group. Third, HbA1c was increased significantly in the SDB PAP group compared to the SDB untreated group.

Our finding that PAP treatment was not associated with a significant change in median weight and BMI in patients with DM2 is consistent with the findings of previous meta-analyses (15, 18, 35). Feng et al. included in their analysis 128 patients with DM2 who received PAP treatment for a maximum of 4 months (15), Zhu et al. analyzed 496 patients with DM2 from randomized controlled trials (RCTs) with PAP treatment for 6 months or shorter (18). Both concluded that the effect of short-term PAP treatment on BMI was not significant in patients with DM2 (15, 18).

In contrast to these findings, a large meta-analysis with 3,181 patients from 25 RCTs reported a significant increase in weight and BMI following OSA treatment with PAP (23). With the exception of one study (36), PAP use ranged from 1–6 months with a median duration of 3 months (23). All of the mentioned meta-analyses investigated the short-term effects of PAP on weight and BMI. In contrast, the median duration of PAP use in our analysis was 1.6 years, representing a long-term PAP application.

Ou et al. analyzed the long-term effect of PAP on weight and BMI in a study with 2,843 patients (30% had DM2) and found no difference in weight change between the PAP and the control group after a mean follow-up of 3.8 years (37). A sub-analysis of those patients with DM2 was not performed (37). However, male participants who used PAP ≥4 h per night gained slightly more weight than matched male control subjects without PAP (37).

We did not find an increase in median weight following PAP treatment, such as the meta-analysis of Drager et al. (23). However, we found an increased OR for a severe weight gain ≥5 kg in patients with PAP treatment. Thus, PAP treatment may not result in clinically significant weight gain in all patients, but in some. Supporting this hypothesis, in a large retrospective analysis, the majority of PAP-treated patients with OSA (84%) had no significant change in weight during a 5-year follow-up period (38). However, 10% of the patients gained significant weight during the PAP treatment (38). These weight gainers were the youngest and most obese at baseline and had a higher prevalence of impaired fasting glucose or DM2 (38). Previous studies investigating the long-term effect of PAP on BMI and weight included a proportion of patients with pathological glucose tolerance no >40% (36–39), whereas the present analysis only examined patients with DM2 (Table 5).

**Table 5:** Studies investigating changes in weight and glycemic control following PAP treatment.

**References**	**Study design**	**Number of patients**	**Patients with pathological glucose tolerance**	**PAP duration**	**Results**
Ou et al. (37)	RCT (PAP + standard care vs. standard care)	2,483	30% (DM)	3.8 ± 1.5 years	(i) No difference in weight change between the PAP and control group, in male and female subjects. (ii) Male participants who used PAP ≥ 4 h/night gained slightly more weight than matched male control subjects without PAP.
Barbé et al. (36)	RCT (PAP vs. no active intervention)	723	not given	4 (2.7; 4.4) years	(i) BMI decreased significantly in the control group compared to the PAP group. (ii) There was no change in BMI in the PAP group.
Loffler et al. (40)	Randomized clinical trial (CPAP + usual care vs. usual care alone)	888	31% (DM2) 51% (prediabetes)	4.3 years (range 16 days−6.9 years)	(i) In those with preexisting diabetes and prediabetes, there was no significant difference in HbA1c between the PAP and usual care groups. (ii) In participants with preexisting diabetes who were not using insulin, there was a small increase in HbA1c in PAP users.
Guest et al. (41)	Case-control	300	100% (DM2)	5 years	(i) At baseline, there was no difference in HbA1c between DM2 patients with and without PAP (7.5 vs. 7.4%) (ii) PAP treated patients had a significant lower HbA1c at 5 years follow-up than untreated patients (8.2 vs. 12.1%).
Myllylä et al. (38)	Retrospective	1,023	40% (DM2/impaired fasting glucose)	6.6 ± 1.2 years	(i) 84% of OSA patients had no significant weight change during PAP treatment. (ii) In 10% of OSA patients significant weight gain was observed.
Basoglu et al. (39)	Observational	1,415	15% (DM2)	1.1 ± 1.0 years	(i) PAP is not associated with a significant weight change in OSA patients. (ii) In non-obese patients (BMI <30 kg/m^2^), weight and BMI increased after PAP. (iii) In obese patients (BMI ≥ 30 kg/m^2^), weight and BMI were reduced after PAP.
Malik et al. (42)	Observational	62	100% (DM2)	12 months	(i) 59% of the DM2 patients showed a decline in HbA1c with PAP treatment. (ii) 7% of DM2 Patients showed a significant improvement in HbA1c with PAP.
Schaller et al. this issue	Observational	346	100% (DM2)	1.6 (1.2;2.2) years	(i) PAP is not associated with a significant median weight change. (ii) HbA1c increased significantly in patients with PAP treatment compared to untreated patients. (iii) PAP is associated with an increased odds ratio for a severe weight gain ≥ 5 kg and an increase in HbA1c.

*BMI, body-mass index; DM2, type 2 diabetes mellitus; HbA1c, hemoglobin A1c; OSA, obstructive sleep apnea; PAP, positive airway pressure; RCT, randomized controlled trial*.

In addition to changes in weight, we analyzed changes in glycemic control measured by HbA1c following PAP treatment. In contrast to previous evidence, our analysis showed a significant increase in HbA1c following PAP treatment in patients with DM2. Five meta-analyses have investigated the effect of PAP treatment on HbA1c in patients with DM2, and all of them reported that PAP did not alter HbA1c levels significantly (15–19). However, none of these studies exceeded a follow-up duration of 6 months. This short-term use of PAP might not be long enough to detect changes in HbA1c (18). HbA1c is a measure of glucose levels during the last 2–3 months, which takes time to change (18). A randomized clinical trial investigated the long-term effect of PAP on glycemic control in 888 patients with OSA and stable cardiovascular disease (40). In those with pre-existing DM2 (*n* = 274, 31%) and prediabetes (*n* = 452, 51%), there was no significant difference between the PAP and usual care groups in HbA1c during follow-up (median duration 4.3 years) (40). However, when restricted to patients with pre-existing DM2 but not using insulin, a slight but significant increase in HbA1c in PAP users was found (40). Two other studies examining the association between long-term PAP and glycemic control were controversial (41, 42) (Table 5). Therefore, further studies analyzing the long-term effect of PAP on HbA1c in patients with DM2 are warranted.

Some possible mechanisms could explain how PAP treatment could promote weight gain and an increase in HbA1c. PAP may reduce the basal metabolic rate (43). This could lead to a positive energy balance followed by weight gain since patients with OSA do not change their eating behavior and physical activity after PAP treatment (43, 44). Furthermore, PAP treatment is associated with increased levels of growth hormone and insulin-like growth factor 1 (45–47). The restoration of the growth hormone/insulin-like growth factor 1 axis after PAP treatment may lead to changes in body composition and an increase in lean body mass (47). Growth hormone does not only promote protein anabolism and muscle growth but it is also considered diabetogenic, such as insulin resistance and hyperglycemia (48). This could aggravate existing insulin resistance in patients with DM2 and thus impair glycemic control as observed in the present analysis. There were no significant differences between the groups regarding insulin substitution, oral antidiabetic drugs, or physical activity at baseline or follow-up that would explain the increase in HbA1c within the observation period in the SDB PAP group (Supplementary Table 1).

The association between weight gain, cardiovascular disease, and mortality in patients with DM2 is controversial (49, 50). The observed 3 mmol/mol (0.3%) increase in HbA1c in patients with DM2 and PAP treatment could result in an increased risk of all-cause and cardiovascular mortality since in a previous meta-analysis, every 11 mmol/mol (1%) increase in HbA1c was associated with a 15% increase of all-cause mortality and a 25% increase in cardiovascular mortality in patients with DM2 (51).

The strengths of the present analysis are the sample size and the high-resolution phenotyping of a study sample of outpatients with DM2 in a prospective setting. The observation period of 2.3 (2.2; 2.4) years and the median PAP duration of 1.6 years allowed us to investigate the changes in weight and glycemic control following PAP treatment over a long period of time. Furthermore, the compliance to the PAP treatment was high with a mean usage of 6.2 hours per night.

The following limitations warrant discussion: First, due to the observational character of the study, a causal relationship of the findings in this analysis cannot be concluded. Second, due to the simplified SDB monitoring, we cannot distinguish between obstructive and central sleep apnea. However, a large cohort study with a study sample representative of the German population suggested that the vast majority of patients had OSA and only a minority had central sleep apnea (52). Third, all information regarding the use of a PAP device was collected from self-reported data and not verified by data from the PAP device. It was shown that the objectively measured PAP use was approximately 1 h less than the patients have reported (53). Fourth, no data regarding caloric intake, eating behavior, or psychological traits were assessed. Thus, possible confounders for change in weight and HbA1c are missing in the present analysis.

In conclusion, median weight change was similar in patients with SDB with and without PAP treatment. However, patients with DM2 and PAP treatments have an increased risk of severe long-term weight gain and an increase in HbA1c. Further research on the long-term effect of PAP on weight change and glycemic control in patients with DM2 is warranted.

## Data Availability Statement

The raw data supporting the conclusions of this article will be made available by the authors, without undue reservation.

## Ethics Statement

The studies involving human participants were reviewed and approved by Ethikkommission University Regensburg. The patients/participants provided their written informed consent to participate in this study.

## Author Contributions

LS, SS, and MA were involved in the conception, hypotheses delineation, and design of the present sub-study, the analysis and interpretation of such information, writing the article, and in its revision prior to submission. CB, IH, and BJ are the principal investigators of the DIACORE study and were involved in the design of this sub-study, the acquisition and interpretation of the data, and the critical revision of the article prior to submission. SS is the guarantor of this work and, as such, had full access to all data of the study and takes responsibility for the integrity of the data and the accuracy of the data analysis. All authors contributed to the article and approved the submitted version.

## Funding

The DIACORE study was funded by the KfH-Stiftung Präventivmedizin e.V. CB received funding from the KfH-Stiftung Präventivmedizin e.V., the Else Kröner-Fresenius-Stiftung, and the Doktor Robert Pfleger-Stiftung. The DIACORE-SDB sub-study was funded by ResMed (Martinsried, Germany). The authors declare that this study received funding from KfH-Stiftung Präventivmedizin e.V., ResMed, and the Doktor Robert Pfleger-Stiftung. The funders were not involved in the study design, collection, analysis, interpretation of data, the writing of this article or the decision to submit it for publication.

## Conflict of Interest

MA received research grants from ResMed, the ResMed Foundation, Philips Respironics, and the Else Kröner-Fresenius-Foundation (2018_A159) as well as lecture and consulting fees from ResMed, Philips Respironics, Boehringer-Ingelheim, NRI, Novartis and Bresotec. The remaining authors declare that the research was conducted in the absence of any commercial or financial relationships that could be construed as a potential conflict of interest.

## Publisher's Note

All claims expressed in this article are solely those of the authors and do not necessarily represent those of their affiliated organizations, or those of the publisher, the editors and the reviewers. Any product that may be evaluated in this article, or claim that may be made by its manufacturer, is not guaranteed or endorsed by the publisher.

## Abbreviations

AHI: apnea-hypopnea-index
BMI: body-mass index
DIACORE: DIAbetes CohORtE
DM2: type 2 diabetes mellitus
HbA1c: hemoglobin A1c
OSA: obstructive sleep apnea
PAP: positive airway pressure
SDB: sleep-disordered breathing
WHR: waist-to-hip ratio.
