# Supplementary material for: Long-Term Weight Change and Glycemic Control in Patients With Type 2 Diabetes Mellitus and Treated vs. Untreated Sleep-Disordered Breathing—Analysis From the DIAbetes COhoRtE
Source: Front Neurol. 2021 Dec 2;12:745049. doi: 10.3389/fneur.2021.745049 (PMC8675635; doi:10.3389/fneur.2021.745049)
Supplement: Supplementary file 1 [file Table_1.PDF]

Supplementary Table 1. Differences between the SDB PAP and SDB untreated groups in antidiabetic medication, insulin substitution, physical activity and excessive daytime sleepiness at baseline and follow-up visit.

	**SDB PAP**	**SDB untreated**	**p value**
n (%)	60 (17.3)	286 (82.7)	
Baseline oral antidiabetic drugs, n (%)	48 (80.0)	215 (75.2)	0.426
Follow-up oral antidiabetic drugs, n (%)	45 (75.0)	208 (72.7)	0.718
Baseline insulin substitution, n (%)	18 (30.0)	109 (38.1)	0.236
Follow-up insulin substitution, n (%)	26 (43.3)	119 (41.6)	0.806
Baseline low physical activity, n (%)	36 (60.0)	164 (57.3)	0.705
Follow-up low physical activity, n (%)	32 (53.3)	153 (53.7)	0.960
Baseline excessive daytime sleepiness, n (%)	4 (6.7)	18 (6.3)	0.900
Follow-up excessive daytime sleepiness, n (%)	4 (6.7)	14 (4.9)	0.587

PAP = positive airway pressure; SDB = sleep-disordered breathing. Excessive daytime sleepiness = Epworth Sleepiness Scale ≥ 11; low physical activity = exercise < 3x/week.
