# Supplementary material for: Long-Term Weight Change and Glycemic Control in Patients With Type 2 Diabetes Mellitus and Treated vs. Untreated Sleep-Disordered Breathing—Analysis From the DIAbetes COhoRtE
Source: Front Neurol. 2021 Dec 2;12:745049. doi: 10.3389/fneur.2021.745049 (PMC8675635; doi:10.3389/fneur.2021.745049)
Supplement: Supplementary file 2 [file Data_Sheet_1.PDF]

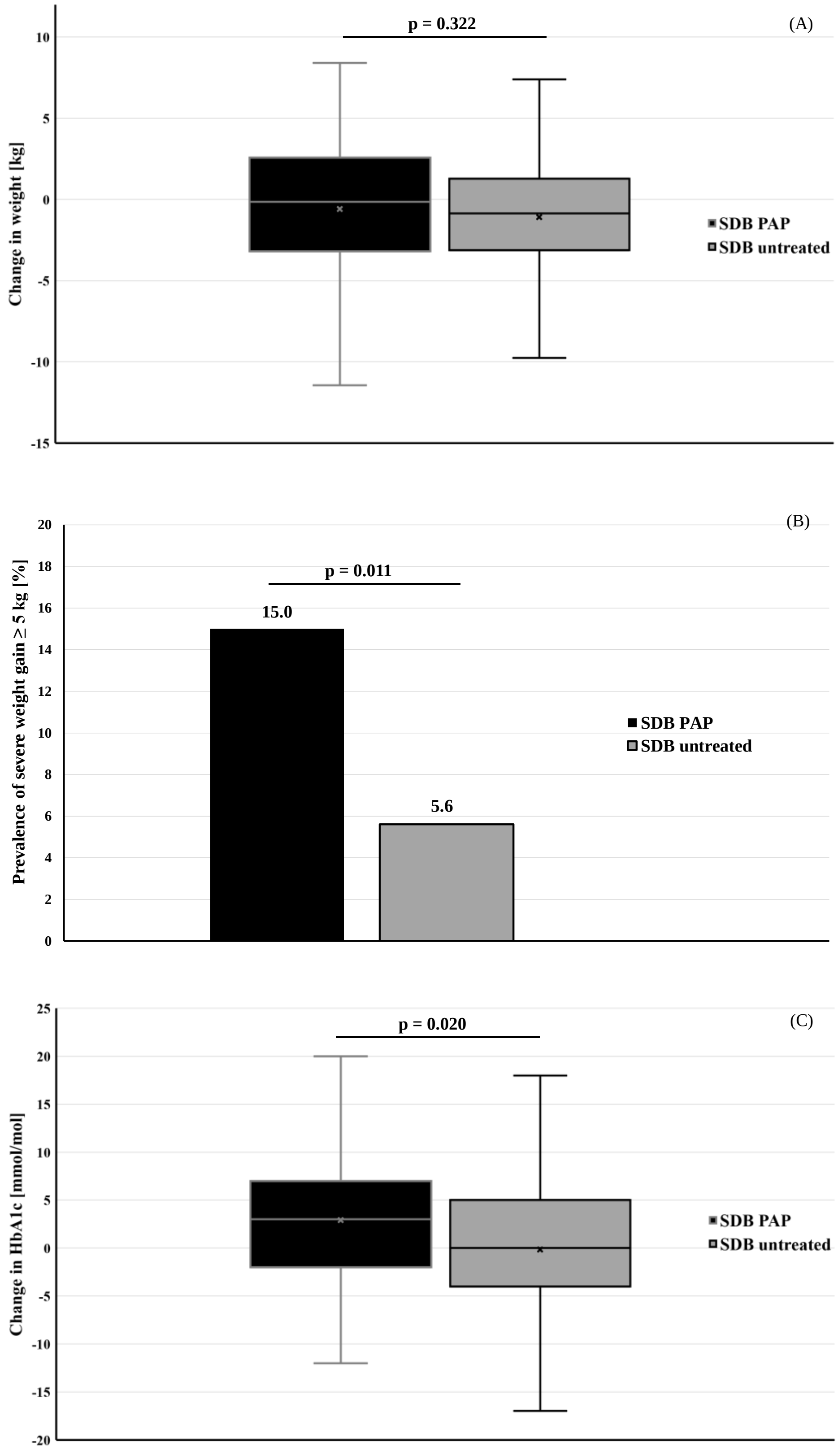

Supplementary Figure 1. (A) Mean weight change was similar in SDB patients with and without PAP treatment. However, patients with type 2 diabetes mellitus and PAP treatment have **(B)** an increased risk of severe long-term weight gain and (**C)** an increase in HbA1c. HbA1c = hemoglobin A1c; PAP = positive airway pressure; SDB = sleep-disordered breathing.
